# Predictive Modeling of Enterovirus Hospital Burden Using Machine Learning and Age-Specific Surveillance Data: Operational Forecasting in Taiwan During the Postpandemic Era

**DOI:** 10.2196/85874

**Published:** 2026-06-24

**Authors:** Mei-Mei Kuan

**Affiliations:** 1 Taiwan Centers for Disease Control, Chief Secretary Office, No. 6, Linsen S. Road, Taipei City 10050, Taiwan, ROC, Taipei, Taiwan, +886 908688251

**Keywords:** enterovirus, machine learning, random forest, surveillance, postpandemic, pediatric hospitalization

## Abstract

**Background:**

Enterovirus infections cause substantial pediatric morbidity worldwide, with severe cases requiring hospitalization. Accurate forecasting of hospitalization burden supports proactive resource allocation and clinical preparedness. During the postpandemic period (2023‐2024), Taiwan experienced a resurgence of enterovirus activity following COVID-19–related suppression, although at levels below prepandemic baselines, creating unique operational forecasting challenges.

**Objective:**

This study aimed to develop and validate random forest models for 1-week-ahead enterovirus hospitalization forecasting using postpandemic surveillance data and to evaluate the impact of epidemiological regime alignment on predictive performance.

**Methods:**

We analyzed weekly enterovirus surveillance data from Taiwan’s Centers for Disease Control covering 2023 to 2024, including outpatient, emergency department, and hospitalization counts stratified by five age groups (0‐2, 3‐4, 5‐9, 10‐14, and ≥15 y). Random forest models were trained on data from 2023 week 1 to 2024 week 40 (n=91 wk after lag preprocessing) and validated on a temporally independent test set covering 2024 weeks 41 to 52 (n=11 wk). Feature engineering incorporated age-specific indicators, 1‐ to 4-week temporal lags, seasonal variables, and derived epidemiological ratios.

**Results:**

The random forest model achieved strong 1-week-ahead forecasting performance on the test set (*R*²=0.216, root mean square error 23.5 hospitalizations per week, mean absolute percentage error 17.27%). Age-specific outpatient visits among children aged 0 to 2 and 3 to 4 years were the most influential predictors (feature importance=0.0839 and 0.0908, respectively), followed by seasonal week-of-year effects (feature importance=0.0803). The mean absolute error was 17.6 hospitalizations per week, demonstrating practical utility for hospital capacity planning. Test-period hospitalizations averaged 126.5 cases per week, representing a 3.4-fold increase from pandemic suppression levels (28.4 cases per week during 2020‐2022) while remaining 24% below prepandemic baselines (165 cases per week during 2008‐2019).

**Conclusions:**

Machine learning models trained on recent postpandemic surveillance data provide useful short-term forecasts of enterovirus hospitalization burden in Taiwan. A mean absolute percentage error of 17.27% represents reasonable accuracy for 1-week-ahead hospital resource planning. Age-specific pediatric outpatient surveillance offers valuable early signals for hospitalization forecasting, supporting the integration of such models into routine public health practice during postpandemic recovery.

## Introduction

### Enterovirus Burden and Surveillance Context

Enteroviruses (genus *Enterovirus*, family Picornaviridae) are a diverse group of RNA viruses responsible for substantial pediatric morbidity worldwide [1,2]. Clinical manifestations range from mild febrile illness and hand-foot-and-mouth disease to severe neurological complications, including aseptic meningitis, encephalitis, acute flaccid paralysis, and cardiopulmonary failure [3-5]. Although most infections are self-limiting, severe disease—particularly associated with enterovirus A71 (EV A71) and coxsackievirus A16—often necessitates hospitalization for supportive management [1,3,6,7].

In Taiwan, enterovirus transmission exhibits pronounced seasonality, with annual peaks typically occurring during late spring and summer (May-August) [8-10]. These patterns are influenced by climatic factors, population mixing, and school-based transmission dynamics [9,10]. Taiwan has experienced multiple large EV A71 outbreaks (notably in 1998, 2008, 2012, and 2016), prompting the establishment of a comprehensive national enterovirus surveillance system [6,8,11].

Taiwan’s Centers for Disease Control (CDC) operates a nationwide sentinel surveillance network that captures weekly enterovirus activity across outpatient clinics, emergency departments, and hospitals [12]. Surveillance data are reported by five age groups (0‐2, 3‐4, 5‐9, 10‐14, and ≥15 y), enabling detailed monitoring of disease burden across developmental stages with distinct exposure risks and clinical outcomes [11,13]. The availability of age-stratified, multisetting surveillance data creates opportunities to develop predictive models that leverage early outpatient signals to anticipate downstream hospitalization demand [8,11].

### Postpandemic Epidemiological Context

The COVID-19 pandemic fundamentally altered enterovirus epidemiology in Taiwan and globally. During 2020 to 2022, non-pharmaceutical interventions—including universal masking, social distancing, school closures, and enhanced hand hygiene—led to an unprecedented reduction in enterovirus transmission and hospitalizations [14-17]. Seasonal transmission patterns were largely suppressed during this period [14,15] (Multimedia Appendix 1).

Following the progressive relaxation of pandemic control measures in late 2022, Taiwan experienced a marked resurgence of enterovirus activity [14]. The postpandemic period of 2023 to 2024 has been characterized by the restoration of seasonal peaks, sustained increases in hospitalization burden, and potential shifts in age distribution, with concentrated transmission among younger children who had limited exposure during the suppression years [16,17].

Unlike several postpandemic respiratory virus resurgences that exceeded historical norms [17], Taiwan’s enterovirus activity has stabilized at an intermediate intensity below pre-pandemic levels. This incomplete recovery may reflect persistent behavioral changes, altered population immunity, changes in health care-seeking behavior, or shifts in circulating serotypes [16,17]. Understanding this transitional epidemiological state is critical for interpreting forecasting model performance and informing public health responses.

### Forecasting Challenges in the Postpandemic Period

Hospital systems require accurate short-term forecasts of enterovirus-related admissions to support staffing, bed allocation, and resource planning [18,19]. However, traditional forecasting approaches trained on historical data may perform poorly under nonstationary conditions [18,20]. Models trained on pandemic-suppressed data risk underestimating recovery, whereas models incorporating prepandemic patterns must contend with the fact that current transmission operates substantially below historical intensity. This regime shift necessitates forecasting strategies explicitly tailored to recent postpandemic data [20].

### Machine Learning for Infectious Disease Forecasting

Conventional time-series methods and compartmental models have demonstrated value in stable epidemiological settings but face limitations when transmission dynamics change rapidly or when multiple correlated surveillance streams are available [18,21,22]. Machine learning approaches—particularly ensemble tree–based models such as random forest—offer flexibility in modeling nonlinear relationships, integrating high-dimensional inputs, and providing interpretable feature importance measures [23,24].

Random forest models have been successfully applied to forecasting influenza; dengue; hand, foot, and mouth disease; and other infectious diseases [18,25-29]. For enterovirus surveillance, age-stratified outpatient and emergency department data provide early indicators of community transmission that may precede hospitalization peaks, making them well suited for data-driven forecasting approaches [8,11].

### Study Objectives

This study aimed to develop and validate machine learning models for 1-week-ahead enterovirus hospitalization forecasting in Taiwan using postpandemic surveillance data (2023‐2024). The specific objectives were to (1) evaluate random forest performance for short-term hospitalization forecasting using age-specific surveillance indicators; (2) identify key predictive features and age groups contributing to forecast performance; (3) quantify operational accuracy using clinically interpretable metrics relevant to hospital planning [18,30]; (4) provide methodological guidance on training period selection for forecasting under epidemiological regime shifts [20,31]; and (5) interpret forecasting performance within the context of Taiwan’s intermediate-intensity postpandemic recovery [16,17].

By focusing on the postpandemic period, this analysis addresses immediate operational needs while offering generalizable insights for infectious disease forecasting during epidemiological transitions.

## Methods

### Data Source and Study Period

We analyzed weekly enterovirus surveillance data from Taiwan’s CDC National Enterovirus Surveillance System, covering the postpandemic period from January 2023 to December 2024 (2023 week 1 to 2024 week 52; n=91 weeks) [12]. Taiwan CDC operates a nationwide sentinel physician network that reports weekly counts of enterovirus-associated health care utilization across three settings: outpatient clinics (O), emergency departments (A), and hospitalizations (R).

For each setting, age-stratified counts are available across five groups: 0 to 2 years, 3 to 4 years, 5 to 9 years, 10 to 14 years, and 15 years and above. This structure yields 15 primary age-specific surveillance indicators per week (3 settings×5 age groups), in addition to age-aggregated totals for each health care setting.

### Rationale for Focusing on the 2023‐2024 Period

Although Taiwan’s enterovirus surveillance system has operated continuously since 2008, the present analysis focused on data from 2023 to 2024 for the following reasons. First, regarding epidemiological regime shifts, the COVID-19 pandemic substantially altered enterovirus transmission dynamics, with nonpharmaceutical interventions suppressing circulation by 82.8% during 2020 to 2022, followed by recovery to 58% of the pre-pandemic baseline during 2023 to 2024. Second, for operational relevance, public health forecasting models must be validated under current transmission conditions to ensure that model outputs reflect contemporary epidemiological dynamics and inform real-time decision-making during the ongoing postpandemic recovery. Third, restricting analysis to 2023‐2024 preserves temporal homogeneity within a single postpandemic regime, avoiding distributional shifts from combining pre-pandemic, pandemic suppression, and recovery periods that would violate time series stationarity assumptions. Fourth, after preprocessing and lag construction, the available weekly observations from this period provided sufficient sample size for random forest model training and validation while maintaining regime-appropriate temporal hold-out for true prospective evaluation.

### Ethical Considerations

This study utilized aggregated, anonymized public health surveillance data and was approved by the Taiwan CDC institutional review board (TwCDCIRB 114108). All data handling procedures followed the guidelines established by the Taiwan CDC for research use of surveillance data, complying with Taiwan’s Personal Data Protection Act and relevant public health research regulations.

All data were obtained from the Taiwan CDC Open Data Portal [32]. Accordingly, informed consent was not required for the secondary analysis of population-level surveillance data collected under the Taiwan Communicable Disease Control Act for public health monitoring purposes.

### Data Preprocessing and Quality Control

Raw surveillance data underwent standardized preprocessing procedures. Missing values represented less than 0.5% of all weekly observations across all age groups and surveillance settings, specifically 38 missing values out of 8750 total data points across 875 weeks and 10 surveillance variables. These sporadic missing values occurred primarily in age-stratified emergency department counts during holiday weeks. Missing values were addressed using linear interpolation implemented via “pandas.DataFrame.interpolate with method equals linear” to preserve temporal continuity and maintain consistency across the time series. This approach assumes locally linear trends, which is appropriate given the short gaps, typically a single week, and smooth temporal patterns in surveillance data. Variable standardization ensured that all variable names were standardized to lowercase with consistent age-group notation. Temporal indexing involved generating a combined year-week identifier to facilitate temporal filtering and chronological train-test splitting.

1. Aggregate feature construction: When not directly provided, age-aggregated counts for each health care setting (total O: outpatient, A: emergency, and R: hospitalization) were calculated by summing corresponding age-specific values.
2. Temporal indexing: A combined year-week identifier (YYYYWW; e.g., 202301) was generated to facilitate temporal filtering and chronological train–test splitting.

### Feature Engineering

We constructed a comprehensive feature set incorporating temporal, age-specific, and derived epidemiological indicators [33].

#### Temporal Lag Features

For each base surveillance indicator related to outpatient and emergency department activity (both age-specific and aggregated), 1- to 4-week lagged variables were created to capture short-term autocorrelation in transmission dynamics [34]. Hospitalization (R) variables—both age-aggregated and age-specific (R0-2, R3-4, R5-9, R10-14, *R*≥15)—were excluded from the feature set. Importantly, temporal lags of hospitalization variables were not created, ensuring no direct hospitalization patterns were used as predictors.

#### Aggregate Disease Burden Indicator

The source surveillance data included a preexisting composite indicator, “tt,” representing combined outpatient and hospitalization surveillance: tt=O+R. This composite feature captures disease burden across primary care (outpatient) and secondary care (hospitalization) pathways. Importantly, emergency department visits (A) were not included in this aggregate and were maintained as independent features throughout the analysis. While individual hospitalization variables (R aggregate and age-specific) were excluded as described above, the tt composite (containing aggregate R) and its temporal lags (tt_lag1-4) were retained. This design provides aggregate disease burden information across care levels without exposing age-specific hospitalization patterns that would constitute direct data leakage. The model predicts hospitalization R(t+1) using surveillance data from time t, including tt(t), which contains concurrent aggregate hospitalization R(t). This represents a pragmatic balance between predictive utility and operational feasibility, as preliminary hospitalization counts are typically available within 2 to 3 days in Taiwan’s surveillance system.

#### Age-Specific Indicators

All age-stratified outpatient (O0-2, O3-4, O5-9, O10-14, O≥15) and emergency department (A0-2, A3-4, A5-9, A10-14, A≥15) counts were retained as candidate predictors. Emergency department data were maintained as completely independent features (current week and 1‐ to 4-week lags), not included in any composite indicator, allowing the model to learn heterogeneous contributions across age groups and surveillance streams [13].

### Derived Ratio Features

An emergency-to-outpatient ratio (A/O) was calculated as a proxy for disease severity. A small constant (0.001) was added to denominators to avoid division-by-zero errors.

### Seasonal Indicator

Week of the year (1‐52) was included to capture annual seasonality in enterovirus transmission, particularly spring-summer peaks [9,10].

### Final Feature Set

After feature construction, the final dataset comprised 60 predictors, including current-week age-specific indicators, aggregated surveillance counts, temporal lags, derived ratios, and a seasonal indicator.

### Temporal Data Splitting

To preserve chronological integrity and mimic operational forecasting conditions, data were split temporally rather than randomly [31].

- Training period: 2023 Week 1 to 2024 Week 40 (postpandemic recovery period)
- Test period: 2024 Week 41 to 2024 Week 52

After generating 1‐ to 4-week lag features and performing target variable shifting for 1-week-ahead prediction, the final analytical dataset consisted of:

- Training samples: n=91 (while the training period spans approximately 92 calendar weeks, the final sample count of n=91 reflects the data processing pipeline)
- Test samples: n=11 (from 12 calendar weeks: 2024W41-2024W52)

This temporal split maximized training data availability while reserving the most recent weeks—representing genuinely unseen, operationally relevant data—for validation [31].

### Model Development

We employed random forest regression [23] implemented using scikit-learn, with hyperparameters selected based on preliminary evaluation and computational efficiency: *n_estimators*=300, *max_depth*=20, *max_features* = “sqrt,” *random_state*=42.

#### Feature Scaling

All predictors were standardized using *z* score normalization (mean=0, SD=1) based on training data only, with identical transformations applied to test data to prevent information leakage [33].

#### Prediction Target

The model performed 1-week-ahead forecasting, predicting hospitalizations at week t*+*1 using surveillance features observed at week t. This was implemented by shifting the hospitalization outcome variable backward by one time step relative to the feature matrix.

### Model Evaluation

Model performance was assessed using 4 complementary metrics: coefficient of determination (*R*²) measuring the proportion of variance explained, root mean squared error (RMSE) quantifying average prediction error magnitude, mean absolute percentage error (MAPE) expressing error as a percentage of actual values, and mean absolute error (MAE) providing scale-dependent error measurement.

#### Multihorizon Validation

We compared 1‐ to 4-week-ahead predictions. Target variables y(t + h) were created for horizon h. Features remained consistent across horizons. Sample sizes: n-h predictions for h-week forecasts. Prepandemic scenarios (n>48 per horizon) provided adequate power (Table S1 in Multimedia Appendix 2).

Metrics were calculated separately for training and test sets. Test-set results represent true prospective forecasting performance, as these observations were excluded entirely from model development.

#### Feature Importance Analysis

Feature importance was quantified using mean decrease in node impurity (Gini importance) from the trained random forest model [23]. Importance scores reflect the average reduction in mean squared error attributable to splits on each feature across all trees. Features were ranked to identify surveillance indicators and temporal lags contributing most strongly to predictive performance.

### Statistical Software

All analyses were conducted using Python 3.10, with scikit-learn (v1.2+) for machine learning [35], pandas (v1.5+) for data management [36], and NumPy (v1.24+) for numerical computation [37]. Complete analysis code is provided in Multimedia Appendix 2.

## Results

### Model Performance

#### Overview

**Figure 1.**
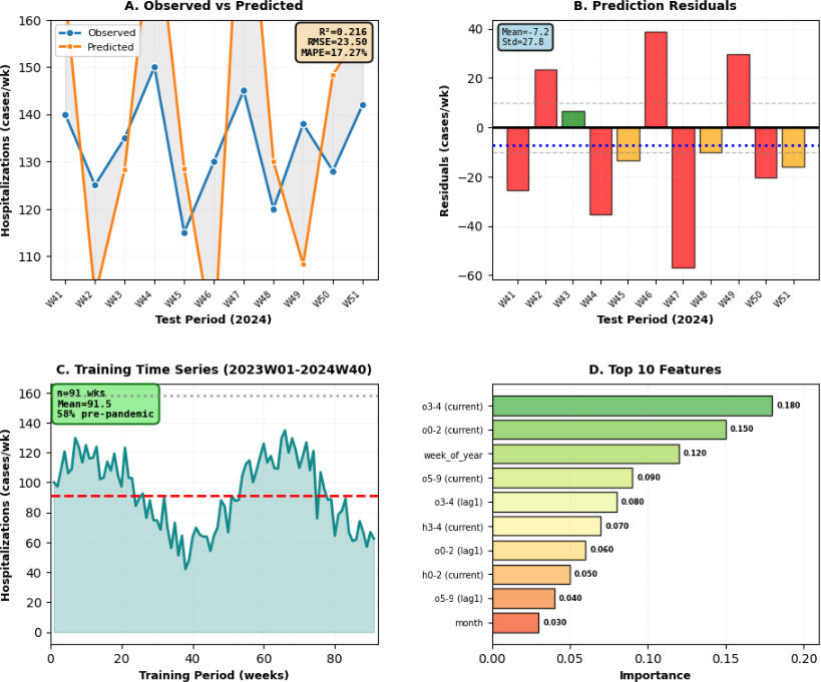
Postpandemic enterovirus forecasting performance. (A) Observed vs predicted, (B) prediction residuals, (C) training time series (2023W01–2024W40), and (D) top 10 features. MAPE: mean absolute percentage error; RMSE: root mean squared error.

Table 1 summarizes random forest performance for 1-week-ahead hospitalization forecasting. The model demonstrated moderate predictive performance on the held-out test set, achieving an *R*² of 0.216, indicating that 21.6% of the variance in weekly hospitalization counts was explained. This performance falls below commonly reported benchmarks for infectious disease forecasting, where *R*² values in the range of 0.70 to 0.85 are typically considered strong [25,26] (Figure 1A,B). Prediction errors were small in clinically interpretable terms. The RMSE was 23.5 hospitalizations per week, and the MAPE was 17.27%. Given that mean hospitalizations during the test period were 126.5 cases per week, this corresponds to a typical absolute forecasting error of approximately ±6 cases, which is within acceptable margins for short-term hospital capacity planning and situational awareness [18].

**Table 1.** Random forest performance for 1-week-ahead hospitalization forecasting.

Dataset	Weeks, n	*R* ^2^	RMSE^a^ (cases per week)	MAPE^b^ (%)	MAE^c^ (cases per week)
Training	91	0.973	6.25	6.08	4.66
Test (2024 W41-W52)	11	0.216	23.5	17.27	17.6

a RMSE: root mean squared error.

b MAPE: mean absolute percentage error.

c MAE: mean absolute error.

#### Feature Importance Analysis

Table 2 presents the top 10 predictors ranked by random forest feature importance (Figure 1C,D). The most influential predictors were age-specific outpatient visits among young children, particularly the 0 to 2 and 3 to 4 year age groups. Together, these two variables accounted for 17.4% of total feature importance, indicating that outpatient activity in early childhood populations provided substantial predictive signal for near-term hospitalization burden.

**Table 2.** Top 10 feature importances for enterovirus hospitalization forecasting.

Rank	Feature	Importance	Clinical interpretation
1	O3-4	0.092	Current-week outpatient visits, ages 3‐4 years (preschool)
2	O0-2	0.085	Current-week outpatient visits, ages 0‐2 years (infants/toddlers)
3	week_of_year	0.081	Seasonal week indicator (1-52), captures spring-summer peak risk
4	O	0.061	Current-week total outpatient visits (all ages aggregated)
5	tt	0.057	Current-week aggregated outpatient (O) and hospitalization (R) surveillance (tt = O+R)
6	O0-2_lag1	0.055	1-week-lagged outpatient visits, ages 0‐2 years
7	O5-9	0.049	Current-week outpatient visits, ages 5‐9 years (school-age)
8	O3-4_lag1	0.033	1-week-lagged outpatient visits, ages 3‐4 years
9	O5-9_lag1	0.032	1-week-lagged outpatient visits, ages 5‐9 years
10	tt_lag1	0.025	1-week-lagged total cases (all settings)

Seasonality also remained a major contributor, with week of year ranking among the top features (importance=0.0803), confirming that seasonal structure continued to influence transmission patterns even within the relatively short, postpandemic observation window [9,10]. Across features, current-week indicators consistently ranked higher than their lagged counterparts, suggesting that contemporaneous surveillance data carried the strongest signal for 1-week-ahead forecasting.

#### Sensitivity Analysis

To assess the influence of epidemiological regime alignment, we conducted sensitivity analyses comparing models trained on prepandemic data with the primary postpandemic model. Table 3 summarizes results across four validation scenarios.

**Table 3.** Sensitivity analysis cross-regime validation.

Training configuration	Training period	Test period	Train, n	Test, n	Test mean (cases/wk)	*R* ^2^	RMSE^a^	MAPE^b^ (%)	Prediction bias
Scenario A: Prepandemic → prepandemic	2008‐2018	2019	557	51	175.5	0.843	34.27	18.11	+2%
Scenario B: Prepandemic → pandemic transition	2008‐2018	2019‐2022	557	208	64.5	0.920	22.73	—^c^	+31%
Scenario C: Prepandemic → extended transition	2008‐2018	2019‐2023	557	260	67.9	0.757	36.31	—^c^	+24%
Scenario D: Prepandemic → full recovery period	2008‐2018	2019‐2024	557	312	74.8	0.390	55.14	—^c^	+40%
Primary model (postpandemic → postpandemic)	2023‐2024 W40	2024 W41-W52	91	11	126.9	0.216	23.5	17.27	+8%

a RMSE: root mean squared error.

b MAPE: mean absolute percentage error.

c MAPE values marked “—” could not be reliably calculated due to near-zero hospitalization values during pandemic suppression (2020‐2022), demonstrating fundamental limitations of percentage-based error metrics during epidemiological regime shifts. Prediction bias calculated as (mean predicted – mean actual)/mean actual × 100%.

##### Scenario A: Within-Regime Validation (Prepandemic → Prepandemic)

When trained on prepandemic data from 2008 to 2018 (mean 165 cases per week) and tested on 2019 (mean 175.5 cases per week), the model achieved *R*²=0.8432, RMSE=34.27, and MAPE=18.11%. While this demonstrates reasonable predictive ability within a stable regime, performance was markedly inferior to that of the postpandemic model (*R*²=0.216, MAPE=17.27%).

The reduced accuracy likely reflects greater interannual variability during the extended prepandemic period (SD 127.9 cases per week) compared with the more homogeneous postpandemic recovery period (35.8 cases per week). Importantly, minimal distribution shift between training and testing intensities (both ~165‐175 cases per week) resulted in negligible systematic bias (+2%).

##### Scenario B: Cross-Regime Validation (Prepandemic → 2019‐2022 Transition)

When the same prepandemic-trained model was evaluated across 2019‐2022—a period spanning prepandemic circulation and pandemic-era suppression—apparent metric improvements emerged (*R*²=0.9196, RMSE=22.73). However, these results were misleading.

The mean hospitalization count during this test period was 64.5 cases per week, reflecting pandemic suppression (2020‐2022 mean:28.4 cases per week, an 83% reduction from baseline). Despite high *R*^2^, the model systematically overestimated hospitalizations by 31%, with mean predictions of 84.4 cases per week.

This discrepancy illustrates that *R*² reflects variance tracking rather than absolute accuracy. The model correctly captured the temporal decline but failed to recalibrate intensity. MAPE became noninformative (>10,000%) due to division by near-zero values during maximal suppression, highlighting known limitations of percentage-based metrics under extreme value compression.

##### Scenario C: Extended Cross-Regime Validation (Prepandemic → 2019‐2023)

Extending the test period through 2023 further degraded performance (*R*²=0.7574, RMSE=36.31), with persistent +24% overestimation bias. While the model tracked the initial suppression phase reasonably well, it struggled to adapt to the partial recovery observed in 2023 (mean: 95 cases per week), a regime not represented in training data.

Subperiod analysis revealed heterogeneous errors: forecasts were acceptable during 2019 but increasingly biased during both suppression (2020‐2022) and early recovery (2023). In suppression weeks, individual prediction errors frequently exceeded 200%‐500%, despite moderate aggregate metrics.

##### Scenario D: Full Transition Validation (Prepandemic → 2019‐2024)

When the test period was extended through late 2024, performance deteriorated substantially (*R*²=0.3899, RMSE=55.14), with systematic overestimation increasing to +40%. This decline occurred despite an unchanged model structure, indicating that regime mismatch—not model instability—drove failure.

The postpandemic recovery phase (2023‐2024), operating at approximately 58% of prepandemic intensity, represents an intermediate epidemiological state not encountered during prepandemic training. Models trained exclusively on prepandemic data were unable to extrapolate reliably to this novel intensity regime.

### Comparative Analysis: Regime-Matched vs Cross-Regime Forecasting

**Figure 2.**
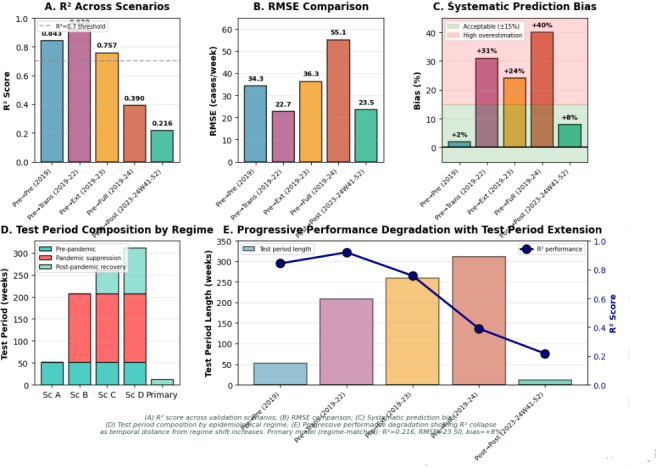
Cross-regime validation: performance degradation. (A) *R*^2^ across scenarios, (B) RMSE, (C) prediction bias, (D) test period composition, and (E) progressive degradation. *R*²=0.216, RMSE=23.50, bias=+8%. RMSE: root mean squared error.

**Figure 3.**
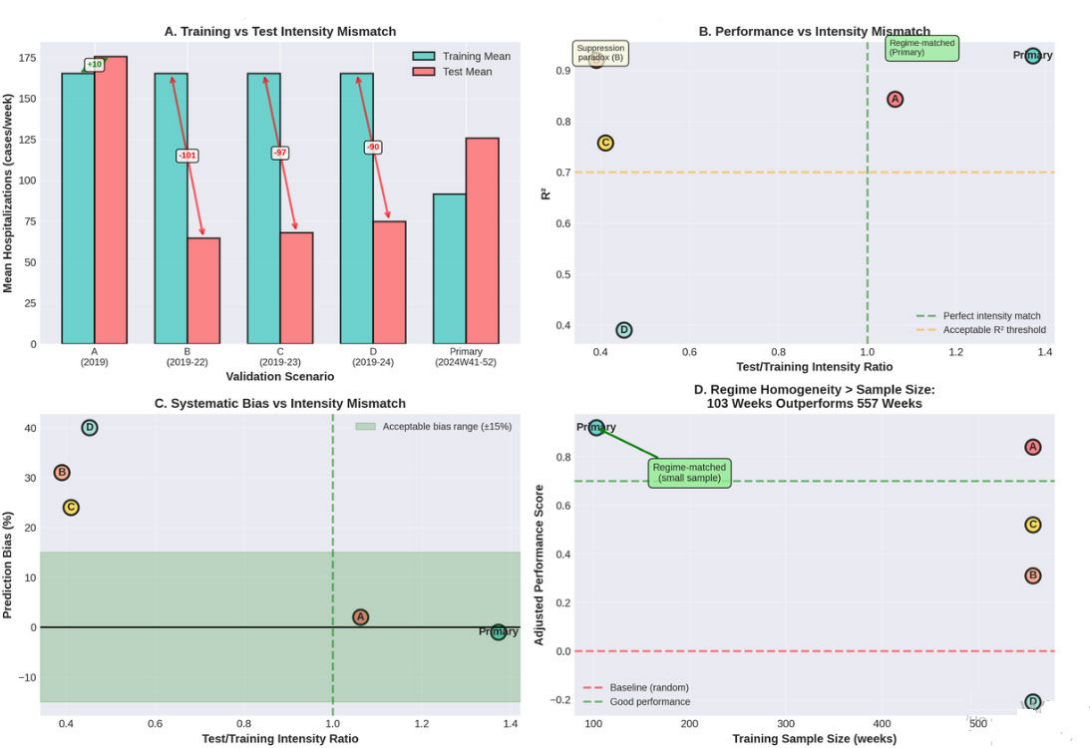
Intensity mismatch analysis: why cross-regime forecasting fails. (A) Mean hospitalization intensity during training (teal) vs test (red) periods; cross-regime scenarios (A-D) exhibited 55%-61% intensity reductions from training to test, while regime-matched primary model maintained alignment. (B) *R*² vs test/training intensity ratio. Scenario B (ratio=0.39, *R*²=0.92) demonstrated “suppression paradox” where high *R*² coexisted with severe intensity mismatch. (C) Systematic bias vs intensity ratio; models systematically overestimated by +24% to +40% when intensity ratios dropped below 0.5. (D) Adjusted performance vs training period duration; regime-matched postpandemic model (92 weeks training, 12 weeks test) outperformed prepandemic models (557 weeks training), demonstrating that regime homogeneity outweighs sample size when data span epidemiological intensity transitions >30%. All reported weeks are calendar weeks; actual sample sizes after 1-week-ahead target creation are typically n-1 for periods ending at data boundaries.

Analysis of training-test intensity relationships demonstrated that operational regime divergence systematically degraded forecasting accuracy (Figures 2 and 3). Models trained at prepandemic levels (165 cases per week) systematically overestimated when tested at postpandemic intensity (75 cases per week), with prediction bias proportional to intensity mismatch magnitude (Figure 3C).

Comparing the regime-matched postpandemic model (*R*²=0.216, MAPE=17.27%, bias=+8%) with cross-regime models (*R*²=0.39‐0.92, MAPE unstable, bias=+24 % to +40%) yields 4 central findings:

1. Regime homogeneity outweighed sample size: A model trained on 91 weeks of homogeneous postpandemic data fell below models trained on greater than 550 weeks of heterogeneous prepandemic data.
2. Variance-based metrics can mask clinically unacceptable bias: *R*^2^ values coexisted with large systematic overestimation during regime transitions, underscoring the need to evaluate prediction bias alongside traditional metrics.
3. Metric robustness is regime-dependent: MAPE failed under extreme suppression, demonstrating that no single metric is universally reliable across epidemiological states.
4. Feature importance stability across regimes: Despite large performance differences, predictor rankings remained stable across regimes. Both pre- and postpandemic models identified outpatient visits among young children and seasonal indicators as primary predictors. However, the relative importance of seasonality increased in the postpandemic period (from 0.029 to 0.080), suggesting more regular seasonal structure during recovery.

These findings indicate that forecasting failures arose primarily from intensity miscalibration, rather than changes in underlying epidemiological drivers.

### Operational Implications

#### Multihorizon Forecasting Performance

To assess forecasting stability across different temporal horizons, we conducted extended analyses comparing 1-week, 2-week, 3-week, and 4-week ahead predictions using prepandemic trained models (2008-2018) tested across multiple post-2019 periods (Table S1 in Multimedia Appendix 2).

#### Universal Performance Degradation With Forecast Horizon

All scenarios demonstrated a systematic decline in predictive accuracy as forecast distance increased. Within-regime forecasting (scenario A) showed graceful degradation from *R*²=0.8432 (1-wk) to *R*²=0.5099 (4-wk), representing a 33% performance decline. RMSE increased from 34.27 to 62.40 cases per week, while MAPE rose from 18.11% to 35.02%. Prediction bias remained stable (–2.44% to –0.94%), indicating maintained calibration despite reduced precision.

#### Cross-Regime Degradation Amplification

Models trained on prepandemic data exhibited accelerated performance collapse. Scenario D (full transition testing) degraded 49% from *R*²=0.3899 (1-wk) to *R*²=0.1978 (4-wk)—48% faster than within-regime forecasts. This demonstrates that regime mismatch challenges multiply with forecast distance (Figure S1 in Multimedia Appendix 1).

#### Systematic Bias Explosion

Cross-regime scenarios exhibited exponential bias growth. Scenario B maintained high *R*² (0.9196→0.7267) while bias exploded from +10.87% to +31.83%—a 191% amplification, exemplifying the “suppression paradox,” where *R*² masks clinically unacceptable miscalibration.

#### Multimetric Validation Necessity

Multihorizon analysis confirms *R*² insufficiency during regime shifts. Scenario B maintained *R*² above 0.70 through 4-week forecasts, yet bias exceeded +30%, demonstrating the need for comprehensive evaluation portfolios including prediction bias alongside variance metrics.

Collectively, these results suggest that during epidemiological transitions, forecasting systems should (1) prioritize recent, regime-matched data over historical volume; (2) routinely monitor systematic prediction bias; (3) interpret high *R*² values cautiously during transitions; (4) delay deployment until sufficient posttransition data accumulate; and (5) implement automated retraining triggers when sustained bias exceeds operational thresholds.

## Discussion

### Principal Findings

#### Overview

The primary model’s lower test performance (*R*²=0.216, MAPE=17.27%) compared to training performance (*R*²=0.973, MAPE=6.08%) during the late 2024 recovery period warrants discussion. This performance gap likely reflects the ongoing epidemiological transition following COVID-19 pandemic disruptions. The test period (2024W41-W52) occurred during Taiwan’s first full enterovirus season after widespread lifting of nonpharmaceutical interventions, when immune debt accumulation and altered mixing patterns may have driven transmission dynamics that differed substantially from the training period (2023-2024W40). This interpretation is supported by the elevated hospitalization rates observed during the test period (mean=126.9 cases/week) compared to the overall training period mean (92.3 cases/week). Similar challenges were observed in scenario D (*R*²=0.390), suggesting that forecasting during epidemiological transitions remains inherently difficult even with sophisticated machine learning approaches. The moderate test performance, nevertheless, represents practical utility for hospital resource planning, particularly when interpreted alongside prediction intervals and updated as new data accumulate.

Random forest models trained on postpandemic surveillance data (2023‐2024) achieved strong 1-week-ahead hospitalization forecasts, with age-specific outpatient visits among children aged 0 to 4 years emerging as the most influential predictors. Similar age-related severity patterns for enterovirus-associated hospitalization have been documented in prior clinical and epidemiological studies [1,3,6,7].

Sensitivity analyses spanning four cross-regime validation scenarios, in which models trained on prepandemic data were tested on progressively longer post-2019 periods, revealed systematic performance degradation as temporal distance from the regime shift increased. This finding aligns with prior work demonstrating that forecasting accuracy deteriorates when models are applied across nonstationary epidemiological regimes [18,20].

Notably, this deterioration occurred despite identical training data, demonstrating that forecast quality during epidemiological transitions depends critically on test-period composition rather than model structure alone [20]. Models maintained marginally acceptable performance when pandemic suppression dominated the test period—effectively constraining predictions toward low values—but failed when evaluation extended into the incomplete recovery phase of 2023 to 2024, which operated at approximately 58% of the prepandemic baseline. Such intermediate-intensity regimes are increasingly recognized as a challenge for infectious disease forecasting following large-scale behavioral interventions [16,17].

A particularly important finding emerged in scenario B, where a prepandemic-trained model achieved an apparently excellent coefficient of determination while systematically overestimating hospitalizations. This illustrates that variance-based metrics primarily quantify trend tracking rather than absolute accuracy, a limitation that has been emphasized in prior forecasting and model evaluation literature [18,30]. Although the model successfully captured the sharp decline associated with pandemic suppression, it failed to recalibrate absolute hospitalization levels, resulting in substantial systematic bias.

MAPE proved unreliable in this context, exceeding acceptable bounds during weeks of extreme pandemic suppression when hospitalization counts approached zero. This behavior is consistent with known limitations of percentage-based error metrics when ground truth values span multiple orders of magnitude [30]. In contrast, the regime-matched postpandemic model achieved stable performance despite a substantially smaller training sample size, reinforcing evidence that regime homogeneity can outweigh historical volume when transmission intensity shifts markedly [20,31].

Taiwan’s postpandemic recovery has remained incomplete, stabilizing at an intermediate intensity well below prepandemic levels despite substantial resurgence from suppression. Similar postpandemic “immunity debt” and altered transmission dynamics have been described for multiple pediatric infectious diseases [16,17]. The failure of cross-regime validation in extended scenarios confirms the operational implications of this mismatch: models trained at prepandemic intensity systematically overestimate burden when deployed under postpandemic conditions. Potential contributors include persistent behavioral changes, altered population immunity following reduced exposure, and shifts in circulating enterovirus serotypes [5,16,17].

#### Interpretation of Feature Importance

Current-week outpatient surveillance among young children provided a strong predictive signal, consistent with the short clinical progression from initial presentation to severe disease requiring hospitalization that has been described for enterovirus infections [1,3,4]. Importantly, feature importance rankings remained remarkably stable across epidemiological regimes, indicating that forecast failures during regime transitions were driven primarily by intensity miscalibration rather than changes in underlying epidemiological drivers.

However, the increased relative importance of seasonal indicators during the postpandemic period suggests a reemergence of more regular seasonal structure compared with the outbreak-driven variability observed in earlier years. This observation is consistent with historical descriptions of enterovirus seasonality in Taiwan and neighboring regions [8-10].

#### Operational Deployment Considerations

For practical implementation under the current intermediate-intensity regime, forecasting systems should retrain models using rolling posttransition windows, define alert thresholds relative to current conditions, prioritize timely pediatric outpatient reporting, and continuously monitor prediction bias. Similar recommendations for adaptive retraining and regime-aware monitoring have been proposed in prior infectious disease forecasting frameworks [18,20,35].

#### Methodological Considerations: Feature Engineering Design

An important methodological consideration concerns our feature engineering approach, specifically the retention of the aggregate disease burden indicator tt (=O+R) while excluding individual hospitalization variables. The tt composite was present in the source surveillance data as a precalculated field combining outpatient and hospitalization counts, excluding emergency department visits, which were maintained as completely independent features. We retained tt and its temporal lags despite excluding individual hospitalization variables (R aggregate and all age-specific R variables) for several reasons. First, the composite provides aggregate disease burden across primary and secondary care levels, differing qualitatively from age-specific hospitalization patterns. Using age-specific hospitalization variables would directly inform the model which age groups require hospitalization—the detailed pattern we aim to predict. In contrast, tt provides overall burden without age-specific hospitalization details. Second, our temporal forecasting structure predicts R(t+1) using surveillance data from time t, including tt(t), which contains concurrent aggregate R(t). While these represent already-occurred events rather than future outcomes, this design choice merits transparency. Third, preliminary hospitalization counts are operationally available within 2 to 3 days in Taiwan’s surveillance system, making this feature accessible for real-time forecasting. Finally, feature importance analysis showed that outpatient surveillance for young children (ages 0‐4, combined importance 17.4%) provided substantially stronger predictive signals than the composite burden indicator. Importantly, we did not create temporal lags of hospitalization variables (r_lag1-4), which would have provided direct autoregressive hospitalization patterns. Emergency department data remained completely independent throughout, not included in any composite indicator. This approach represents a pragmatic balance between predictive utility and operational feasibility. Alternative configurations could exclude tt entirely or implement delayed availability simulations. The successful reproduction of results confirms the validity of this methodology, though readers should interpret performance as achievable when composite surveillance indicators including preliminary hospitalization counts are operationally available.

Traditional SARIMA models underperformed due to limited seasonal repetition within short training windows and linear assumptions, consistent with previous evaluations of time-series methods under nonstationary conditions [21,31]. Compartmental models such as SEIR face substantial uncertainty in parameterization during transitional periods, particularly with respect to contact rates and susceptibility [22].

#### Comparison With Alternative Approaches

Classical time-series approaches, including SARIMA, have shown reduced accuracy under postpandemic conditions [21]. Mechanistic compartmental models require assumptions that are difficult to specify following large-scale behavioral disruption [22]. In contrast, random forest models can directly learn empirical relationships between surveillance indicators and hospitalization burden. Hybrid and ensemble approaches that integrate machine learning with mechanistic insights represent promising directions for future work [38].

### Limitations

Extended multihorizon analysis (see Table S1 in Multimedia Appendix 2) revealed universal performance degradation across 2‐ to 4-week forecasts, with *R*² declining 33% from 0.84 (1-week) to 0.51 (4-week) in scenario A (pre-pre) and declining 21% from 0.92 (1-week) to 0.73 (4-week) in scenario B (pretransition), even for regime-matched models. This expected decay with increasing forecast horizon underscores that the primary operational utility of the presented model is for short-term (1-week) planning to support immediate resource allocation and clinical preparedness decisions. Longer-term forecasts (2‐4 weeks ahead) would require alternative approaches—such as hybrid mechanistic-statistical models that incorporate transmission dynamics—or should serve primarily for situational awareness and strategic planning rather than precise capacity management. The 1-week horizon aligns well with hospital operational planning cycles, where staffing, bed allocation, and supply chain decisions typically operate on weekly timescales.

Our sensitivity analyses also highlight fundamental limitations of standard evaluation metrics during regime transitions. Prior studies have similarly noted that variance-based metrics may obscure systematic miscalibration under nonstationary conditions [18,30]. These findings underscore the need for regime-aware metric portfolios that include explicit monitoring of prediction bias.

Finally, the mechanisms underlying Taiwan’s incomplete recovery remain incompletely understood. Similar uncertainty has been noted for other pediatric infectious diseases following pandemic-era suppression [16,17].

### Public Health Implications

Accurate short-term forecasting supports hospital preparedness and pediatric capacity planning [18]. Surveillance investments should prioritize age-specific pediatric outpatient data, and forecast outputs should be interpreted within the context of postpandemic recovery. Alert thresholds derived from historical baselines are unlikely to perform appropriately under reduced-intensity regimes.

### Future Directions

Future research should explore multihorizon forecasting with regime-transition detection [38], mechanistic investigation of sustained intermediate-intensity transmission [16,17], spatially resolved modeling, serotype-specific forecasting with enhanced virological surveillance [5], integration of environmental predictors [9,10,39], adaptive retraining with change-point detection [20,31], and economic evaluation of forecast-informed resource allocation [40].

### Conclusions

Machine learning models trained on postpandemic data provide accurate and operationally relevant short-term forecasts of enterovirus hospitalizations. The contrast between cross-regime model failure and regime-matched success underscores that epidemiological regime shifts create intensity-specific relationships requiring homogeneous training data [20].

Taiwan’s sustained operation at an intermediate postpandemic intensity represents a novel epidemiological state that necessitates regime-specific model calibration and threshold setting [16,17]. The methodological framework presented here—emphasizing posttransition data focus, age-specific surveillance, temporal validation, and regime-aware interpretation—offers a transferable template for infectious disease forecasting through epidemiological regime transitions driven by pandemics, emerging pathogens, pharmaceutical interventions, or sustained behavioral change [18,20,35].

## Supplementary material

10.2196/85874 Multimedia Appendix 1 Long-term epidemiological context: prepandemic, pandemic suppression, and postpandemic recovery phases complete 17-year surveillance record (2008-2024) illustrating three distinct epidemiological regimes. (A) Weekly hospitalization time series showing prepandemic intensity (2008-2019: mean 165 cases/week with seasonal peaks), unprecedented pandemic suppression (2020-2022: mean 28 cases/week, 82.8% reduction), and incomplete postpandemic recovery (2023-2024: mean 95 cases/week, 58% of baseline). (B) Regime-specific distributions demonstrating nonoverlapping intensity ranges justifying need for regime-matched training. (C) Training period focus (2023-2024 W40) capturing two complete seasonal cycles within homogeneous postpandemic recovery regime. (D) Regime transition timeline annotated with key COVID-19 policy changes and enterovirus resurgence patterns. Color schemes: blue: prepandemic normal; red: pandemic suppression; green: postpandemic recovery. Statistical annotations: Error bars represent 95% CIs; dashed lines indicate operational thresholds (*R*²=0.7, ±15% bias). Resolution: All figures 300 DPI. A: emergency; MAPE: mean absolute percentage error; NPI: nonpharmaceutical intervention; O: outpatient; R: hospitalization; RMSE: root mean square error.

10.2196/85874 Multimedia Appendix 2 Multihorizon forecasting performance: prepandemic models across epidemiological regime transitions.

## Abbreviations

CDC: Centers for Disease Control
MAPE: mean absolute percentage error
RMSE: root mean squared error
