# Supplementary figures and images for: Predictive Modeling of Enterovirus Hospital Burden Using Machine Learning and Age-Specific Surveillance Data: Operational Forecasting in Taiwan During the Postpandemic Era
Source: JMIR Form Res. 2026 Jun 24;10:e85874. doi: 10.2196/85874 (PMC13292981; doi:10.2196/85874)

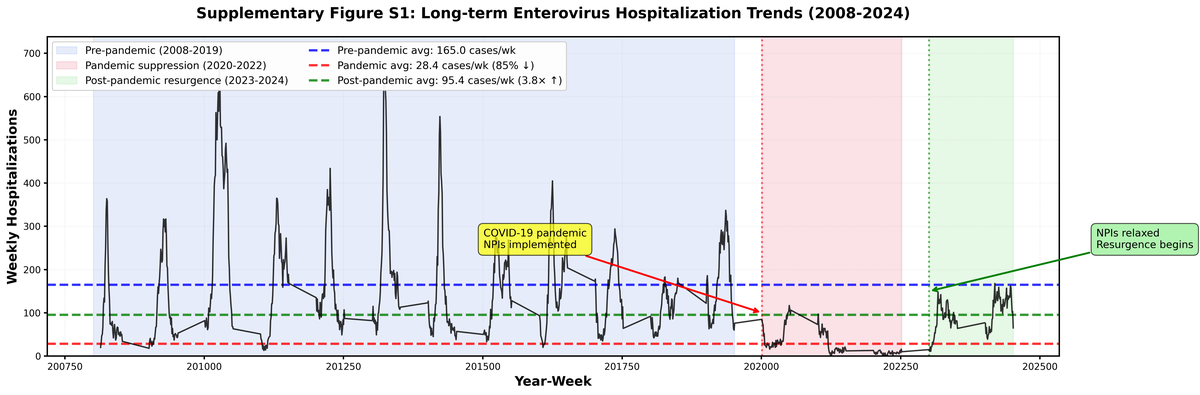

Supplement: Multimedia Appendix 1 [file formative-v10-e85874-s001.png]
