# Supplementary material for: Predictive Modeling of Enterovirus Hospital Burden Using Machine Learning and Age-Specific Surveillance Data: Operational Forecasting in Taiwan During the Postpandemic Era
Source: JMIR Form Res. 2026 Jun 24;10:e85874. doi: 10.2196/85874 (PMC13292981; doi:10.2196/85874)
Supplement: Multimedia Appendix 2 [file formative-v10-e85874-s002.docx]

Supplementary Materials

**Table S1. Multihorizon Forecasting Performance: Prepandemic Models Across Epidemiological Regime Transitions**

| Training Configuration | Test Period | Test Mean (cases/week) | Horizon | N | R² | RMSE | Bias (%) | Notes |
| --- | --- | --- | --- | --- | --- | --- | --- | --- |
| Scenario A: Within-Regime (Pre→Pre) | 2019 (52 weeks) | 175.5 | 1-week | 51 | 0.8432 | 34.27 | -2.44 | Baseline |
|  |  |  | 2-week | 50 | 0.7437 | 44.25 | -1.10 |  |
|  |  |  | 3-week | 49 | 0.6597 | 51.49 | -0.74 |  |
|  |  |  | 4-week | 48 | 0.5099 | 62.40 | -0.94 |  |
|  |  |  | Degradation |  | -33% | +82% | Stable | Graceful |
| Scenario B: Cross-Regime (Pre→Trans) | 2019-2022 (209 weeks) | 64.5 | 1-week | 208 | 0.9196 | 22.73 | +10.87 | R² paradox |
|  |  |  | 2-week | 207 | 0.8707 | 28.75 | +17.05 |  |
|  |  |  | 3-week | 206 | 0.8203 | 33.78 | +22.70 |  |
|  |  |  | 4-week | 205 | 0.7267 | 41.64 | +31.83 |  |
|  |  |  | Degradation |  | -21% | +83% | +191% | Bias explosion |
| Scenario C: Extended (Pre→2023) | 2019-2023 (261 weeks) | 67.9 | 1-week | 260 | 0.7574 | 36.31 | +24.06 | Accelerating |
|  |  |  | 2-week | 259 | 0.6616 | 42.80 | +30.51 |  |
|  |  |  | 3-week | 258 | 0.5621 | 48.53 | +36.23 |  |
|  |  |  | 4-week | 257 | 0.4330 | 55.18 | +44.66 |  |
|  |  |  | Degradation |  | -43% | +52% | +86% | Severe |
| Scenario D: Maximal (Pre→2024) | 2019-2024 (313 weeks) | 74.8 | 1-week | 312 | 0.3899 | 55.14 | +40.09 | Catastrophic |
|  |  |  | 2-week | 311 | 0.3031 | 58.84 | +44.82 |  |
|  |  |  | 3-week | 310 | 0.2658 | 60.24 | +47.42 |  |
|  |  |  | 4-week | 309 | 0.1978 | 62.94 | +51.93 |  |
|  |  |  | Degradation |  | -49% | +14% | +29% | Collapse |

*Note:* Training period (2008-2018) had mean 165.0 cases/week across 557 weeks. All scenarios used identical Random Forest architecture (n_estimators=300, max_depth=20) with 1-4 week lagged age-specific surveillance features. Degradation calculated as (4-week - 1-week) / 1-week × 100%. MAPE values omitted for Scenarios B-D due to near-zero pandemic suppression weeks. N indicates number of test predictions available at each horizon. Bias calculated as (mean predicted - mean actual) / mean actual × 100%.

## Table S2 Legend

**Background.** To evaluate forecasting robustness across temporal horizons and epidemiological regime transitions, we trained Random Forest models on pre-pandemic surveillance data (2008-2018, mean 165 cases/week) and tested predictions at 1-4 week horizons across four scenarios spanning pre-pandemic stability (A), pandemic suppression (B), incomplete recovery (C), and full transition (D).

**Scenario A (Within-Regime Baseline).** Pre-pandemic training and testing (2008-2018 → 2019) established baseline degradation patterns. Performance degraded gracefully (-33% R²) with stable low bias (<3%), demonstrating that extended horizons challenge forecasting even within homogeneous contexts due to signal attenuation.

**Scenario B (Suppression Paradox).** Testing across 2019-2022 pandemic transition revealed apparently excellent R² (0.92→0.73, only -21% degradation) coexisting with explosive bias (+11%→+32%, +191% amplification). Models tracked variance patterns but systematically miscalibrated absolute predictions, validating our core finding that R² measures trend tracking, not prediction accuracy, during regime transitions.

**Scenario C (Incomplete Recovery).** Extended testing through 2023 (mean 67.9 cases/week, 41% of pre-pandemic) showed accelerating degradation (-43% R²). Front-loaded degradation (1→2 week: -13%) followed by continued erosion suggests regime mismatch manifests immediately and compounds progressively.

**Scenario D (Maximal Divergence).** Full 2019-2024 testing (mean 74.8 cases/week, 55% below training) demonstrated catastrophic collapse (R²=0.39→0.20, -49%). Four-week forecasts approached random walk performance (R²<0.20), quantifying the amplifier effect of regime mismatch across forecast horizons.

**Key Finding: Cross-Regime Amplification.** Comparing within-regime (-33%) versus maximal cross-regime (-49%) revealed 48% faster performance collapse when training-operational intensities diverge >50%. This quantifies the multiplier effect: challenges at 1-week amplify exponentially rather than linearly with temporal distance.
